# Steady Periodic Hydroelastic Waves in Polar Regions

**DOI:** 10.1007/s42286-024-00095-1

**Published:** 2024-06-06

**Authors:** Bogdan-Vasile Matioc, Emilian I. Părău

**Affiliations:** 1 Fakultät für Mathematik, Universität Regensburg, 93040 Regensburg, Germany; 2 School of Mathematics, University of East Anglia, Norwich, NR4 7TJ UK

**Keywords:** Hydroelastic waves, Local bifurcation, Rotational waves, 76B15, 74F10, 35B32

## Abstract

We construct two-dimensional steady periodic hydroelastic waves with vorticity that propagate on water of finite depth under a deformable floating elastic plate which is modeled by using the special Cosserat theory of hyperelastic shells satisfying Kirchhoff’s hypothesis. This is achieved by providing a necessary and sufficient condition for local bifurcation from the trivial branch of laminar flow solutions.

## Introduction

Hydroelastic waves propagate in polar regions at the surface of water covered by a deformable ice sheet. The water is modeled as an inviscid and incompressible fluid with constant density (set to be 1). Assuming that the flow is two-dimensional, the equations of motion are the Euler equations

1.1a $$\begin{aligned} \left. \begin{array}{rllllll} &{}&{} u_t+ u u_x+ vu_y = -P_x, \\[1ex] &{}&{} v_t+ uv_x+ vv_y = -P_y-g, \\[1ex] &{}&{} u_x+v_y = 0, \end{array} \right\} \qquad -d<y<\eta (t,x), \end{aligned}$$

where *u* is the horizontal velocity, *v* is the vertical velocity, *P* is the pressure, and *g* is the gravitational acceleration. The fluid domain is bounded from below by a flat impermeable bed located at $y=-d$, where $d>0$ is a constant, and the free wave surface $\{y=\eta (t,x)\}$ is assumed to be a thin ice sheet which is modeled as a thin elastic plate using the Cosserat theory of hyperelastic shells satisfying Kirchhoff’s hypothesis [21, 31]. The inertia of this thin elastic plate is neglected, we assume that the plate is not pre-stressed, and consider only the effect of bending, neglecting the stretching of the plate. Therefore, we impose the following boundary conditions

1.1b $$\begin{aligned} \left. \begin{array}{rlllllll} P&{}=&{}\alpha H(\eta )&{} \quad \text {on } y=\eta (t,x),\\[1ex] v&{}=&{}\eta _t +u\eta _x&{}\quad \text {on } y=\eta (t,x),\\[1ex] v&{}=&{}0&{}\quad \text {on } y=-d, \end{array} \right\} \end{aligned}$$

where $\alpha >0$ is a constant,

$$\begin{aligned} H(\eta ):=\omega (\eta )^{-1}\big [\omega (\eta )^{-1}\big (\omega (\eta )^{-3}\eta _{xx}\big )_{x}\big ]_x+\frac{1}{2}\big (\omega (\eta )^{-3}\eta _{xx}\big )^3, \end{aligned}$$

and

1.1c $$\begin{aligned} \omega (\eta ):=(1+\eta _x^2)^{1/2}. \end{aligned}$$

We investigate herein the existence of periodic steady water wave solutions to (1.1a)–(1.1c) for which the unknowns $u,\, v,\, P,\, \eta$ satisfy

$$\begin{aligned} (u,v,P)(t,x,y)= (u,v,P)(x-ct,y) \qquad \text {and}\qquad \eta (t,x)= \eta (x-ct), \end{aligned}$$

where $c>0$ is the speed of the wave. Moreover, letting $\lambda >0$ denote the period of the wave, we restrict to solutions which fulfill

1.1d $$\begin{aligned} \int _0^\lambda \eta (x)\, \textrm{d}x=0. \end{aligned}$$

Setting

$$\begin{aligned} \Omega _\eta :=\{(x,y)\in {\mathbb {R}}^2:\, -d<y<\eta (x)\}, \end{aligned}$$

we, thus, look for functions $u,\, v,\, P:\overline{\Omega _\eta }\rightarrow {\mathbb {R}}$ and $\eta :{\mathbb {R}}\rightarrow {\mathbb {R}}$ which are $\lambda$-periodic in *x* and solve (after replacing $u-c$ by *u*)^1^ the coupled system of equations

1.2a $$\begin{aligned} \left. \begin{array}{rllllll} &{}&{} u u_x+ vu_y = -P_x&{}\quad \text {in } \, \Omega _\eta , \\[1ex] &{}&{} uv_x+ vv_y = -P_y-g&{}\quad \text {in }\, \Omega _\eta , \\[1ex] &{}&{} u_x+v_y = 0&{}\quad \text {in } \, \Omega _\eta ,\\[1ex] &{}&{} P = \alpha H(\eta )&{}\quad \text {on } \, y=\eta (x),\\[1ex] &{}&{} v = u\eta '&{}\quad \text {on } \, y=\eta (x),\\[1ex] &{}&{} v = 0&{}\quad \text {on } \, y=-d,\\ &{}&{} \displaystyle \int _0^\lambda \eta (x)\, \textrm{d}x = 0. \end{array} \right\} \end{aligned}$$

We also exclude the presence of stagnation pointy by requiring that

1.2b $$\begin{aligned} u<0\quad \text {in } \overline{\Omega _\eta }. \end{aligned}$$

A similar setting has been considered in [20] where, using a variational approach, the authors establish the existence of hydroelastic solitary waves for sufficiently large values of the dimensionless parameter $\alpha$ under the assumption that the vorticity is zero. Within the same irrotational scenario, the authors of [2] establish the existence of symmetric envelope hydroelastic solitary waves by using spatial dynamics techniques. Moreover, in [3, 4], the existence of periodic hydroelastic waves which may posses a multi-valued height between two superposed irrotational fluid layers with positive densities separated by an elastic plate was shown via global bifurcation theorem, the analysis being based on the reformulation of the problem as a vortex sheet problem.

We also mention the paper [10] where, in the rotational setting, weak periodic solutions to a related problem which accounts also for surface tension effects at the free boundary are constructed via a variational approach. These weak solutions are minimizers of the total energy per period functional among flows subject to three constraints—the volume of fluid per period, the circulation per period on the water surface, and the rearrangement class of the vorticity field—and are subsequently shown to have the property that the vorticity is a decreasing function of the stream function. The approach in [10] is different from the one we choose where we fix from the start the vorticity function, which is not assumed to be monotonic, together with the volume of fluid for period and the relative mass flux to construct classical solutions to the hydroelastic wave problem (1.2) via local bifurcation theory.

Hydroelastic wave models which allow for both bending and stretching of the elastic wave surface have been studied in the context of flows without vorticity in [8, 32].

The initial-value problem for flexural-gravity waves has been investigated in [6] by developing a well-posedness theory based on a vortex sheet formulation. Related local well-posedness results art established in [25] where also inertial effects for the floating elastic plate are included and in [36] in the setting of hydroelastic waves with vorticity in dimension $n\ge 2$.

For numerical studies of hydroelastic waves, in the setting of constant vorticity, we refer to the recent works [17, 18, 35].

In the present paper, we extend the existence theory for (1.2) by allowing for a general vorticity 

$$\begin{aligned} {\overline{\omega }}:=u_y-v_x. \end{aligned}$$

The vorticity is a very important aspect of ocean flows also in polar regions, a non-zero vorticity characterizing waves that interact with non-uniform currents such as the Antarctic Circumpolar Current or near-surface currents in the Arctic Ocean, see, e.g., [1, 12, 28, 33]. An essential tool in our analysis is the availability of two equivalent formulations of (1.2), the stream function formulation (2.2) and the height function formulation (2.3), see Proposition 2.1. In particular, the condition (1.2b) enables us to introduce the so-called vorticity function ${\gamma :[p_0,0]\rightarrow {\mathbb {R}}}$, where the constant $p_0<0$ is the relative mass flux, which determines, via the stream function formulation (2.2), the vorticity ${\overline{\omega }}$ of the flow, see Sect. 2. While we consider a general Hölder continuous vorticity function $\gamma$, in order to establish the existence (and uniqueness) of a laminar flow solution to (1.2) (with a flat wave profile located at ${y=0}$ and *x*-independent velocity and pressure) the restriction (1.3) is required on $\gamma$ and the physical parameters. This laminar flow solution is a solution to (1.2) for each value of the wavelength $\lambda$. We will then use $\lambda$ as a bifurcation parameter in order to determine other nonlaminar symmetric (with respect to the horizontal line $x=0$) hydroelastic waves. It turns out that bifurcation can occur if and only if a second condition, see (1.4), is satisfied. In the setting of irrotational waves these conditions are explicit, see Remark 1.3. To prove our main result in Theorem 1.1, we cannot directly use the aforementioned formulations (2.2) or (2.3) of the problem because the boundary condition in these formulations that corresponds to the dynamic boundary condition (1.2a)_4_ involves fourth order derivatives of the unknown, whereas the elliptic equation posed in the (fluid) domain is of second order. However, inspired by an idea used also in other steady water wave problems, see [7, 16, 23, 27, 29, 30, 34], we may reformulate (2.3), after rescaling the horizontal variable by $\lambda$, as a quasilinear elliptic equation subject to a boundary condition which may be viewed as a compact, but at the same time nonlocal and nonlinear, perturbation of the trace operator, see (3.8). The wavelength $\lambda$ appears as a free parameter in (3.8) and we show that the local bifurcation theorem of Crandall and Rabinowitz, cf. [14, Theorem 1.7], can be applied in the context of (3.8) to prove our main result and establish in this way the existence of solutions to (1.2) within the regularity class introduced in Proposition 2.1.

### Theorem 1.1

Let $\alpha >0$, $d>0$, $p_0<0,$ and $g>0$ be fixed and choose $\beta \in (0,1)$. Assume that the vorticity function $\gamma$ belongs to $\textrm{C}^\beta ([p_0,0])$ and set

$$\begin{aligned} \Gamma (p):=\int _{p_0}^p\gamma (s)\,\textrm{d}s,\qquad p\in [p_0,0]. \end{aligned}$$

Then, we have:

(i) The problem (1.2) has laminar solutions $(u,\, v,\, P,\,\eta )=(u_*,v_*,P_*,0)$, with ${u_*,\,v_*,\,P_*}$ independent of the *x*-variable, iff
  1.3 $$\begin{aligned} \lim _{\vartheta \searrow 2\underset{[p_0,0]}{\max }\Gamma } \int _{p_0}^0(\vartheta -2\Gamma (s))^{-1/2}\textrm{d}s>d. \end{aligned}$$
  If (1.3) is satisfied, there exists exactly one laminar solution $(u_*,v_*,P_*,0)$ to (1.2).
(ii) Assume that the condition (1.3) holds true and set $a:=(\vartheta -2\Gamma )^{1/2}\in \textrm{C}^{1+\beta }([p_0,0]),$ where $\vartheta >2\,\underset{[p_0,0]}{\max }\,\Gamma$ is the unique constant which satisfies
  $$\begin{aligned} \int _{p_0}^0(\vartheta -2\Gamma (s))^{-1/2}\textrm{d}s=d. \end{aligned}$$
  Then:
  - If
    1.4 $$\begin{aligned} g \int _{p_0}^0\frac{1}{a^{3}(p)}\textrm{d}p< 1 \end{aligned}$$
    does not hold, there exist no solutions to (1.2) which bifurcate from the trivial branch of laminar flow solutions $\{(\lambda , u_*,v_*,P_*,0):\, \lambda >0\};$
  - If (1.4) is satisfied, there exists a unique minimal wavelength $\lambda _*>0$ with the property that $(\lambda _*, u_*,v_*,P_*,0)$ is a local bifurcation point (of the trivial branch) of solutions to (1.2). More precisely, there exists a local bifurcation curve
    $$\begin{aligned} \mathcal {C}=\{(\lambda (s), u(s),v(s),P(s),\eta (s)):\, s\in (-\varepsilon ,\varepsilon )\}, \end{aligned}$$
    where $\varepsilon >0$ is a small constant, having the following properties:
    $[s\mapsto \lambda (s)]$ is smooth, $\lambda (s)>0$ for $s>0,$ and $\lambda (s)=\lambda _*+O(s)$ for $s\rightarrow 0$;
    $$(u(0),v(0),P(0),\eta (0))=(u_*,v_*,P_*,0)$$;
    For $s\ne 0,$ the tupel $(u(s),v(s),P(s),\eta (s))$ is a solution to (1.2) with minimal wavelength $\lambda (s)$ and vorticity function $\gamma$. Moreover, the wave profile has one crest (located on the vertical line $x=0$) and one trough per period, is symmetric with respect to crest and trough lines, and strictly monotone between crest and trough.

Concerning Theorem 1.1, we add the following remarks.

### Remark 1.2

(i) If $0\ne |s|<\varepsilon$, then $(u(s),v(s),P(s),\eta (s))$ is also of solution to (1.2) having (not minimal) period $k\lambda (s)$ for all $1\le k\in {\mathbb {N}}$. In particular, for each $k\ge 1$, $(k\lambda _*, u_*,v_*,P_*,0)$ is also a local bifurcation point of the trivial branch of solutions to (1.2). In Theorem 1.1 we prove that these are the only points on the trivial branch of solutions from where other symmetric solutions bifurcate.
(ii) Our analysis discloses, under the assumption (1.4), that bifurcation from double (actually multiple) eigenvalues of symmetric waves is excluded along the trivial branch of laminar solutions to the hydroelastic waves problem (1.2).

We now illustrate the conditions for bifurcation from Theorem 1.1 in the particular case of irrotational waves (with $\gamma =0$).

### Remark 1.3

If $\gamma =0$, then (1.3) is automatically fulfilled and the inequality (1.4) is equivalent to

$$\begin{aligned} \frac{gd^3}{p_0^2}<1. \end{aligned}$$

Moreover, the wavelength $\lambda _*>0$ can be determined as the unique solution to the equation

1.5 $$\begin{aligned} \left[ g+\alpha \left( \frac{2\pi }{\lambda _*}\right) ^4\right] \tanh \left( \frac{2\pi d}{\lambda _*}\right) =\frac{p_0^2}{d^2}\frac{2\pi }{\lambda _*}. \end{aligned}$$

Equation (1.5) is the dispersion relation for irrotational hydroelastic waves.

In our analysis, we exclude stagnation points, which enables us to use the height function formulation (2.3) and to consider general vorticity functions. However, if stagnation points are present, the stream function formulation (2.2) is still available and for certain classes of vorticity functions (2.2) may be used to construct hydroelastic waves with stagnation points, see, e.g., [15, 23, 26, 30, 34] for different approaches to the classical water wave problem. The global bifurcation problem for (1.2), which could be considered using analytic global bifurcation theory [9], is beyond the goals of this paper.

Outline: In Sect. 2, we present two further equivalent formulations of (1.2): the stream function formulation (2.2) and the height function formulation (2.3). Then, in Sect. 3, we reformulate (2.3) by reexpressing the boundary condition in (2.3) obtained from the dynamic boundary condition as a compact, but nonlinear and nonlocal, perturbation of a Dirichlet boundary condition, see (3.8). Finally, in Sect. 4, we recast (3.8) as a bifurcation problem and prove Theorem 1.1.

## Equivalent Formulations of the Hydroelastic Waves Problem

In this section, we introduce two further equivalent formulations of the hydroelastic waves problem (1.2) which have been useful also when constructing rotational water waves in other physical scenarios, cf., e.g., [11, 22, 24].

### The Velocity Formulation

The stream function $\psi :\overline{\Omega _\eta }\rightarrow {\mathbb {R}}$ is defined by the equations

$$\begin{aligned} \psi =0 \quad \text {on } y=\eta (x)\qquad \text {and}\qquad \nabla \psi =(-v,u)\quad \text {in } \Omega _\eta . \end{aligned}$$

Since $\psi _y<0$, cf. (1.2b), the constant $p_0:=-\psi |_{y=-d}$, called relative mass flux (see [13]) is negative.

Let further $\mathcal {H}:\overline{\Omega _\eta }\rightarrow \overline{\Omega }$, where $\Omega :={\mathbb {R}}\times (p_0,0),$ be defined by the formula

$$\begin{aligned} \mathcal {H}(x,y):=(q(x,y),p(x,y)):=(x,-\psi (x,y)). \end{aligned}$$

As a consequence of (1.2b), the function $\mathcal {H}$ is a bijection. For smooth solutions to (1.2), we then compute

$$\begin{aligned} \partial _q({\overline{\omega }}\circ \mathcal {H}^{-1}) =\left( {\overline{\omega }}_x+\frac{v}{u}{\overline{\omega }}_y\right) \circ \mathcal {H}^{-1}=0\quad \text {in } \Omega , \end{aligned}$$

since (1.2a)_1_–(1.2a)_3_ yield

$$\begin{aligned} u{\overline{\omega }}_x+v{\overline{\omega }}_y=0\quad \text {in } \Omega _\eta . \end{aligned}$$

Hence, there exists a function $\gamma :[p_0,0]\rightarrow {\mathbb {R}}$, the so-called vorticity function, with the property that ${\overline{\omega }}\circ \mathcal {H}^{-1}(q,p)=\gamma (p)$ for all $(q,p)\in \overline{\Omega },$ or equivalently

$$\begin{aligned} {\overline{\omega }}(x,y)=\gamma (-\psi (x,y))\quad \text {for all } (x,y)\in \overline{\Omega _\eta }. \end{aligned}$$

This relation together with (1.2a)_1_–(1.2a)_2_ implies that the energy

$$\begin{aligned} E:=P+\frac{u^2+v^2}{2}+gy-\int _0^\psi \gamma (-s) \, \textrm{d}s \end{aligned}$$

is constant in $\Omega _\eta .$ Evaluating this expression at the wave surface, we deduce together with the relation (1.2a)_4_, that

2.1 $$\begin{aligned} |\nabla \psi |^2+2g\eta +2\alpha H(\eta )=Q\qquad \text {on } y=\eta (x), \end{aligned}$$

where *Q* is a constant. Integration by parts further leads to

$$\begin{aligned} \int _0^\lambda H(\eta )\, \textrm{d}x=0, \end{aligned}$$

and, since also $\eta$ has zero integral mean, we infer from (2.1), after integrating over one period, that

$$\begin{aligned} Q=\frac{1}{\lambda }\int _0^\lambda |\nabla \psi |^2(x,\eta (x))\, \textrm{d}x. \end{aligned}$$

Consequently, $\psi$ solves the boundary value problem

2.2a $$\begin{aligned} \left. \begin{array}{lllllll} &{}&{} \Delta \psi =\gamma (-\psi )&{}\quad \text {in } \,\, \Omega _\eta ,\\[1ex] &{}&{} \psi = 0&{}\quad \text {on } \,\, y=\eta (x),\\[1ex] &{}&{} \psi = -p_0&{}\quad \text {on } \,\, y=-d,\\[1ex] &{}&{} |\nabla \psi |^2+2g\eta +2\alpha H(\eta ) = \displaystyle \frac{1}{\lambda }\int _0^\lambda |\nabla \psi |^2(x,\eta (x)\, \textrm{d}x&{}\quad \text {on } \,\, y=\eta (x) \end{array} \right\} \qquad \end{aligned}$$

and satisfies

2.2b $$\begin{aligned} \psi _y<0\quad \text {in } \, \overline{\Omega _\eta }. \end{aligned}$$

### The Height Function Formulation

We define the height function $h:\overline{\Omega }\rightarrow {\mathbb {R}}$ by

$$\begin{aligned} h(q,p)=y, \end{aligned}$$

which associates with a point $(q,p)\in \overline{\Omega }$ the vertical coordinate of the fluid particle located at ${(x,y)=\mathcal {H}^{-1}(q,p)}\in \overline{\Omega _\eta }$. Then, since $\eta =h(\cdot ,0)$, the function *h* solves the following boundary value problem

2.3a $$\begin{aligned} \left. \begin{array}{rllllll} &{}&{} (1+h_q^2)h_{pp}-2h_ph_qh_{pq}+h_p^2h_{qq}-\gamma h_p^3 = 0&{}\quad \text {in }\,\, \Omega ,\\[1ex] &{}&{} h = -d&{}\quad \text {on } \,\, p=p_0,\\[1ex] &{}&{} \displaystyle \frac{1+h_q^2}{h_p^2}+2gh+2\alpha H(h) = \displaystyle \frac{1}{\lambda }\int _0^\lambda \frac{1+h_q^2}{h_p^2}(q,0) \textrm{d}q&{}\quad \text {on } \,\, p=0, \end{array} \right\} \end{aligned}$$

together with

2.3b $$\begin{aligned} h_p>0\quad \text {in } \overline{\Omega }. \end{aligned}$$

#### Proposition 2.1

(Equivalence of formulations) Let $\beta \in (0,1).$ Then, the following formulations are equivalent:

(i) The velocity formulation (1.2) for
  $$\begin{aligned} u,\,v,\,P\in \textrm{C}^{1+\beta }(\overline{\Omega _\eta }) \text { and } \eta \in \textrm{C}^{4+\beta }({\mathbb {R}}); \end{aligned}$$
(ii) The stream function formulation (2.2) for
  $$\begin{aligned} \psi \in \textrm{C}^{2+\beta }(\overline{\Omega _\eta }), \eta \in \textrm{C}^{4+\beta } ({\mathbb {R}}), \text { and }~{\gamma \in \textrm{C}^{\beta }([p_0,0])}; \end{aligned}$$
(iii) The height function formulation (2.3) for
  $$\begin{aligned} h \in {\hbox {C}}^{2+\beta }(\overline{\Omega }) \text { with } \mathop {\textrm{tr}}\nolimits _0 h\in {\hbox {C}}^{4+\beta }({\mathbb {R}}), \text { and } \gamma \in {\hbox {C}}^{\beta }([p_0,0]). \end{aligned}$$

#### Proof

The proof is similar to that of [13, Lemma 2.1]. $\square$

## An Equivalent Formulation of (2.3)

In our analysis, we will take advantage of the height function formulation (2.3) to establish the existence of steady periodic hydroelastic waves. The main tool used to achieve this goal is the local bifurcation theorem of Crandall and Rabinowitz, cf. [14, Theorem 1.7]. The appropriate parameter for bifurcation is the wavelength $\lambda >0$. Since *h* is $\lambda$-periodic with respect to *q*, it is, therefore, suitable to rescale *h* according to

3.1 $$\begin{aligned} \widetilde{h}(q,p):= h(\lambda q,p),\quad (q,p)\in \overline{\Omega }. \end{aligned}$$

The function $\widetilde{h}$ is 1-periodic and solves (after dropping tildes) the equations

3.2a $$\begin{aligned} \left. \begin{array}{rllllll} &{}&{}(\lambda ^2+h_q^2)h_{pp}-2h_ph_qh_{pq}+h_p^2h_{qq}-\lambda ^2\gamma h_p^3=0&{}\quad \text {in } \,\, \Omega ,\\[1ex] &{}&{} h=-d&{}\quad \text {on } \,\, p=p_0,\\[1ex] &{}&{} \displaystyle \frac{\lambda ^2+h_q^2}{h_p^2}+2g\lambda ^2h+\frac{2\alpha }{\lambda }H \Big (\frac{h}{\lambda }\Big ) = \displaystyle \int _0^1\mathop {\textrm{tr}}\nolimits _0\frac{\lambda ^2+h_q^2}{h_p^2} \, {\textrm{d}}q&{}\quad \text {on }\,\, p=0 \end{array} \right\} \qquad \end{aligned}$$

and

3.2b $$\begin{aligned} h_p>0\quad \text {in } \overline{\Omega }. \end{aligned}$$

In the following, $\mathop {\textrm{tr}}\nolimits _0$ is the trace operator with respect to the boundary component $\{p=0\}$ of $\Omega$, that is, given $f:\overline{\Omega }\rightarrow {\mathbb {R}}$, the function $\mathop {\textrm{tr}}\nolimits _0 f:{\mathbb {R}}\rightarrow {\mathbb {R}}$ is defined by $\mathop {\textrm{tr}}\nolimits _0 f(q)=f(q,0)$ for $q\in {\mathbb {R}}$.

Let $\beta \in (0,1)$ be fixed. We will assume that $h\in \mathbb {X}$, where the Banach spaces $\mathbb {X}$ is defined as follows

$$\begin{aligned} \mathbb {X}:=\left\{ h\in {\hbox {C}}^{2+\beta }(\overline{\Omega }):\, h \text { is even and } 1 \text {-periodic with repect to } q \text { and } \int _0^1\mathop {\textrm{tr}}\nolimits _0h\, \textrm{d}q=0 \right\} . \end{aligned}$$

Similarly, given $k\in {\mathbb {N}}$, the space $\textrm{C}_e^{k+\beta }({\mathbb {R}})$ consists of the even and 1-periodic functions with uniformly $\beta$-Hölder continuous *k*th derivative. Moreover, $\textrm{C}_{e,0}^{k+\beta }({\mathbb {R}})$ is the subspace of ${\textrm{C}_e^{k+\beta }({\mathbb {R}})}$ which contains only functions with zero integral mean. We note that the boundary condition (3.2a)_3_ is not well defined for $h\in \mathbb {X}$ as fourth order derivatives of *h* appear in this equation. However, since of $H(h/\lambda )$ involves only derivatives of *h* with respect to the horizontal variable *q*,  we may reformulate the boundary condition (3.2a)_3_ as a nonlocal and nonlinear compact perturbation of the trace operator $\mathop {\textrm{tr}}\nolimits _0$.

To this end, we set $\zeta :=\mathop {\textrm{tr}}\nolimits _0 h/\lambda$ and note that, if $h\in \mathbb {X}$ satisfies $\mathop {\textrm{tr}}\nolimits _0h\in \textrm{C}_e^{4+\beta }({\mathbb {R}})$, (3.2b), and the boundary condition (3.2a)_3_, then

$$\begin{aligned} H(\zeta )=B(\lambda ,h), \end{aligned}$$

where $B:(0,\infty )\times \{h\in \mathbb {X}\,:\, h_p>0 \text { in } \overline{\Omega }\}\rightarrow \textrm{C}_{e,0}^{1+\beta }({\mathbb {R}})$ is the smooth mapping defined by

3.3 $$\begin{aligned} B(\lambda ,h):=\frac{\lambda }{2\alpha } \left[ \int _0^1\mathop {\textrm{tr}}\nolimits _0\frac{\lambda ^2+h_q^2}{h_p^2}\, \textrm{d}q-\mathop {\textrm{tr}}\nolimits _0\left( \frac{\lambda ^2+h_q^2}{h_p^2} +2g\lambda ^2h \right) \right] . \end{aligned}$$

Integration leads to

$$\begin{aligned} \int _0^x H(\zeta ) \, \textrm{d}s=\int _0^x B(\lambda ,h) \, \textrm{d}s\qquad \text {for all } \,\, x\in {\mathbb {R}}. \end{aligned}$$

In view of the fact that $\mathop {\textrm{tr}}\nolimits _0h$ is an even function, we obtain that

$$\begin{aligned} \int _0^x H(\zeta ) \, \textrm{d}s=\left[ \omega ^{-2}(\zeta )(\omega ^{-3}(\zeta )\zeta '')' +\frac{1}{2}\zeta '\zeta ''^2\omega ^{-7}(\zeta )\right] (x),\qquad x\in {\mathbb {R}}, \end{aligned}$$

where $\omega (\cdot )$ is the nonlinear operator defined in (1.1c), hence

3.4 $$\begin{aligned} \Big [\omega ^{-2}(\zeta )(\omega ^{-3}(\zeta )\zeta '')'+\frac{1}{2}\zeta '\zeta ''^2\omega ^{-7}(\zeta )\Big ](x)=\int _0^x B(\lambda ,h) \, \textrm{d}s\qquad \text { for all } x\in {\mathbb {R}}. \nonumber \\ \end{aligned}$$

Integrating the last relation once more we arrive at

3.5 $$\begin{aligned} \zeta ''(q)= \omega ^{5} (\zeta )(q)\left( C+\int _0^q\int _0^x B(\lambda ,h) \, \textrm{d}s\,\textrm{d}x-\frac{5}{2}\int _0^q \zeta ' \zeta ''^2\omega ^{-7}(\zeta )\, \textrm{d}x\right) \qquad \end{aligned}$$

for all $q\in {\mathbb {R}}$, where $C:=\zeta ''(0).$ Letting $\Phi :(0,\infty )\times \{h\in \mathbb {X}\,:\, h_p>0 \text { in } \overline{\Omega }\}\rightarrow {\hbox {C}}^{1+\alpha }_e({\mathbb {R}})$ be defined by

3.6 $$\begin{aligned} \Phi (\lambda ,h)(q):= & {} \int _0^q\int _0^x B(\lambda ,h) \, \textrm{d}s\,\textrm{d}x \nonumber \\{} & {} -\frac{5}{2}\int _0^q \Big (\frac{\mathop {\textrm{tr}}\nolimits _0 h}{\lambda }\Big )' \Big [\Big (\frac{\mathop {\textrm{tr}}\nolimits _0 h}{\lambda }\Big )''\Big ]^2\omega ^{-7}\Big (\frac{\mathop {\textrm{tr}}\nolimits _0 h}{\lambda }\Big ) \, \textrm{d}x \end{aligned}$$

for $q\in {\mathbb {R}},$ the previous equality identifies, since $\zeta ''$ has zero integral mean, the constant *C* as

$$\begin{aligned} C=-\left( \int _0^1\omega ^{5} (\zeta )\textrm{d}q\right) ^{-1}\int _0^1 \omega ^5(\zeta ) \Phi (\lambda ,h)\,\textrm{d}q, \end{aligned}$$

and, therefore, we have

$$\begin{aligned} (1-\partial _q^2)\zeta =\zeta +\omega ^5(\zeta ) \left( \int _0^1\omega ^{5} (\zeta )\textrm{d}q \right) ^{-1}\int _0^1 \omega ^5(\zeta ) \Phi (\lambda ,h)\textrm{d}q-\omega ^5(\zeta ) \Phi (\lambda ,h)\in \textrm{C}^{1+\alpha }_{e,0}({\mathbb {R}}). \end{aligned}$$

Since $1-\partial _q^2:{\hbox {C}}^{3+\alpha }_{e,0}({\mathbb {R}})\rightarrow \textrm{C}^{1+\alpha }_{e,0}({\mathbb {R}})$ is an isomorphism, we get

$$\begin{aligned} \zeta= & {} (1-\partial _q^2)^{-1}\Big [\zeta +\omega ^5(\zeta ) \Big (\int _0^1\omega ^{5} (\zeta )\,\textrm{d}q\Big )^{-1}\int _0^1 \omega ^5(\zeta ) \Phi (\lambda ,h)\,\textrm{d}q-\omega ^5(\zeta ) \Phi (\lambda ,h)\Big ] \nonumber \\{} & {} \in {\hbox {C}}^{3+\alpha }_{e,0}({\mathbb {R}}). \end{aligned}$$

This proves that $\mathop {\textrm{tr}}\nolimits _0h$ satisfies (3.7) and therewith the first implication in Lemma 3.1 below.

### Lemma 3.1

Let $h\in \mathbb {X}$ satisfy (3.2b). Then, the following are equivalent:

(i) $$\mathop {\textrm{tr}}\nolimits _0h\in \textrm{C}_e^{4+\beta }({\mathbb {R}}) $$ and *h* satisfies (3.2a) _3_;
(ii) With $\Phi$ defined in (3.6) we have
  3.7 $$\begin{aligned} \mathop {\textrm{tr}}\nolimits _0h= & {} (1-\partial _q^2)^{-1} \bigg [\lambda \omega ^5(\mathop {\textrm{tr}}\nolimits _0h/\lambda ) \left( \int _0^1\omega ^{5} (\mathop {\textrm{tr}}\nolimits _0h/\lambda )\,\textrm{d}q \right) ^{-1}\int _0^1 \omega ^5(\mathop {\textrm{tr}}\nolimits _0h/\lambda ) \Phi (\lambda ,h)\,\textrm{d}q \nonumber \\{} & {} +\mathop {\textrm{tr}}\nolimits _0h-\lambda \omega ^5(\mathop {\textrm{tr}}\nolimits _0h/\lambda ) \Phi (\lambda ,h) \bigg ]. \end{aligned}$$

### Proof

It remains to prove that (ii) implies (i). Let, thus, Lemma 3.1 (ii) be satisfied. Then, since the argument of $(1-\partial _q^2)^{-1}$ in (3.7) lies in ${\textrm{C}_{e,0}^{1+\beta }({\mathbb {R}})}$, the function ${\zeta :=\mathop {\textrm{tr}}\nolimits _0 h/\lambda }$ belongs to ${\hbox {C}}^{3+\alpha }_{e,0}({\mathbb {R}})$ and satisfies (3.5). Multiplying now (3.5) by $\omega ^{-5}(\zeta )$ and differentiating the resulting equation once, we deduce that $\zeta$ satisfies the equation (3.4), hence $\zeta \in {\textrm{C}_e^{4+\beta }({\mathbb {R}})}.$ Differentiating (3.4), we deduce that indeed $H(\zeta )=B(\lambda ,h)$, thus (3.2a)_3_ holds true. $\square$

In view of Lemma 3.1, we have formulated the problem (3.2) as the following system

3.8a $$\begin{aligned} \left. \begin{array}{rllllll} &{}&{} (\lambda ^2+h_q^2)h_{pp}-2h_ph_qh_{pq}+h_p^2h_{qq} - \lambda ^2\gamma h_p^3 = 0&{}\quad \text {in } \Omega ,\\[1ex] &{}&{} h = -d\displaystyle &{}\quad \text {on } p=p_0,\\[1ex] &{}&{} h= \Psi (\lambda ,h)&{}\quad \text {on } p=0, \end{array} \right\} \end{aligned}$$

and

3.8b $$\begin{aligned} h_p>0\quad \text {in } \overline{\Omega }, \end{aligned}$$

where $\Psi :(0,\infty )\times \{h\in \mathbb {X}\,:\, h_p>0 \text { in } \overline{\Omega }\}\rightarrow {\hbox {C}}^{3+\alpha }_{e,0}({\mathbb {R}})$ is the smooth mapping given by

3.9 $$\begin{aligned} \Psi (\lambda ,h):= & {} (1-\partial _q^2)^{-1} \Bigg [\lambda \omega ^5(\mathop {\textrm{tr}}\nolimits _0 h/\lambda ) \Bigg (\int _0^1\omega ^{5} (\mathop {\textrm{tr}}\nolimits _0h/\lambda )\,\textrm{d}q\Bigg )^{-1}\int _0^1 \omega ^5(\mathop {\textrm{tr}}\nolimits _0h/\lambda ) \Phi (\lambda ,h)\,\textrm{d}q \nonumber \\{} & {} +\mathop {\textrm{tr}}\nolimits _0h-\lambda \omega ^5(\mathop {\textrm{tr}}\nolimits _0h/\lambda ) \Phi (\lambda ,h)\Bigg ]. \end{aligned}$$

## Local Bifurcation Analysis

In this section, we consider the equivalent formulation (3.8) of the hydroelastic waves problem (1.2) and study its solutions set. In a first step, we investigate in Sect. 4.1 the existence of laminar flow solutions to (3.8). Then, in Sect. 4.2, we formulate (3.8) as a bifurcation problem, see (4.5), and determine a sufficient and necessary condition, see (4.16), for bifurcation from the set of laminar flow solutions. We conclude this section with the proof of the main result.

### Laminar Flow Solutions for (3.8)

We next investigate the existence of laminar flow solutions to (3.8), that is, given $\lambda >0$, we look for solutions $H=H(\lambda )\in \mathbb {X}$ to (3.8) that depend only on the variable *p*. Then, $H\in {\hbox {C}}^{2+\beta }([p_0,0])$ solves the Sturm–Liouville problem

4.1 $$\begin{aligned} \left. \begin{array}{llllll} H'' =\gamma H'^3\qquad \text {in } (p_0,0),\\[1ex] H(p_0)=-d,\quad H(0)=0 \end{array} \right\} \end{aligned}$$

together with the inequality that $H'>0$ in $[p_0,0]$. The next result shows that (1.3) is a sufficient and necessary condition for the existence of a (unique) laminar flow solution.

#### Lemma 4.1

The boundary value problem (4.1) has a solution $H\in \textrm{C}^{2+\beta }([p_0,0])$ with $H'>0$ in $[p_0,0]$ iff (1.3) is satisfied. In this case the solution is unique and it is given by

4.2 $$\begin{aligned} H(p)=-\int _{p}^0 (\vartheta -2\Gamma (s))^{-1/2}\textrm{d}s,\qquad p\in [p_0,0], \end{aligned}$$

where $\vartheta >2\max _{[p_0,0]}\Gamma$ is the unique solution to

4.3 $$\begin{aligned} \int _{p_0}^0(\vartheta -2\Gamma (s))^{-1/2}\textrm{d}s=d. \end{aligned}$$

#### Proof

Since (4.1)_1_ is equivalent to

$$\begin{aligned} \Big (\frac{1}{H'^2}+2\Gamma \Big )'=0, \end{aligned}$$

we obtain that

$$\begin{aligned} \frac{1}{H'^2(p)}=\vartheta -2\Gamma (p),\qquad p\in [p_0,0], \end{aligned}$$

where the constant $\vartheta$ needs to satisfy $\vartheta >2\max _{[p_0,0]}\Gamma$. From the latter relation, we infer that *H* is given by (4.2) and solves (4.1) iff $\vartheta$ is the solution to (4.3). In view of the monotonicity of the integrand in (4.3) with respect to $\vartheta$, the existence of the (unique) solution to (4.3) is equivalent to (1.3). $\square$

### Bifurcation Analysis for (3.8)

In the following, we assume that (1.3) is satisfied and we denote by *H* the laminar flow solution identified in Lemma 4.1. We next define the Banach spaces $\mathbb {Y}$ and $\mathbb {Z}_1\times \mathbb {Z}_2$ consisting of 1-periodic functions with respect to the variable *q* by setting

$$\begin{aligned} \mathbb {Y}:=\{h\in \mathbb {X}\, :\, h=0 \text { on } {p=p_0}\},\qquad \mathbb {Z}_1:=\{h\in {\hbox {C}}^\beta (\overline{\Omega })\,:\, h \text { is even}\},\qquad \mathbb {Z}_2:=\textrm{C}_{e,0}^{2+\beta }({\mathbb {R}}), \end{aligned}$$

and we denote by $\mathcal {O}$ the open subset of $\mathbb {Y}$ defined by

$$\begin{aligned} \mathcal {O}:=\{h\in \mathbb {Y}:\, h_p+H'>0 \quad \text {in } \overline{\Omega }\}. \end{aligned}$$

We further introduce the operator $\mathcal {F}:=(\mathcal {F}_1,\mathcal {F}_2):(0,\infty )\times \mathcal {O}\subset {\mathbb {R}}\times \mathbb {Y}\rightarrow \mathbb {Z}_1\times \mathbb {Z}_2$ by

4.4 $$\begin{aligned} \mathcal {F}_1(\lambda ,h):= & {} (\lambda ^2+h_q^2)(H''+h_{pp}) -2(H'+h_p)h_qh_{pq}+(H'^2+h_p^2)h_{qq}-\lambda ^2 \gamma (H'+h_p)^3, \nonumber \\ \mathcal {F}_2(\lambda ,h):= & {} \mathop {\textrm{tr}}\nolimits _0h-\psi (\lambda ,h+H). \end{aligned}$$

Hence, the problem (3.8) is equivalent to the nonlinear and nonlocal equation

4.5 $$\begin{aligned} \mathcal {F}(\lambda ,h)=0, \end{aligned}$$

where

4.6 $$\begin{aligned} \mathcal {F}\in {\hbox {C}}^\infty ((0,\infty )\times \mathcal {O},\mathbb {Z}_1\times \mathbb {Z}_2) \end{aligned}$$

has the property that

4.7 $$\begin{aligned} \mathcal {F}(\lambda ,0)=0\qquad \text {for all } \lambda >0. \end{aligned}$$

Our goal is to apply the Crandall–Rabinowitz theorem [14, Theorem 1.7] on bifurcation from simple eigenvalues in the context of (4.5) to determine new solutions to (4.5) which are also *q*-dependent. For this reason, we shall determine $\lambda _*>0$ with the property that the partial Fréchet derivative $\partial _h\mathcal {F}(\lambda _*,0)$ is a Fredholm operator of index zero with a one-dimensional kernel.

Given $\lambda >0$, the partial Fréchet derivative $\partial _h\mathcal {F}(\lambda ,0):=(L,T)$ is given by

4.8 $$\begin{aligned} L[h]:= & {} \lambda ^2h_{pp}+H'^2h_{qq}-3\lambda ^2\gamma H'^2h_p, \nonumber \\ T[h]:= & {} \mathop {\textrm{tr}}\nolimits _0 h-(1-\partial _q^2)^{-1}\Bigg [\mathop {\textrm{tr}}\nolimits _0h-\frac{\lambda ^4}{\alpha } \Bigg (S[h]-\int _0^1S[h]\, \textrm{d} q\Bigg )\Bigg ] \end{aligned}$$

for $$h\in \mathbb {Y}$$, where

$$\begin{aligned}S[h](q):=\int _0^q\int _0^x \Bigg [\mathop {\textrm{tr}}\nolimits _0\Bigg (\frac{h_p}{H'^3}- gh\Bigg ) -\int _0^1\mathop {\textrm{tr}}\nolimits _0\frac{h_p}{H'^3} \,\textrm{d}q\Bigg ]\, \textrm{d}s\,\textrm{d}x,\qquad q\in {\mathbb {R}}. \end{aligned}$$

#### Lemma 4.2

Given $\lambda >0$, the Fréchet derivative $\partial _h\mathcal {F}(\lambda ,0)\in \mathcal {L}(\mathbb {Y}, \mathbb {Z}_1\times \mathbb {Z}_2)$ is a Fredholm operator of index zero.

#### Proof

In view of [19, Theorem 6.14], the operator

$$\begin{aligned} (\lambda ^2\partial _p^2+H'^2\partial _q^2,\mathop {\textrm{tr}}\nolimits ):{\hbox {C}}^{2+\beta }(\overline{\Omega })\rightarrow \textrm{C}^{\beta }(\overline{\Omega })\times {\hbox {C}}^{2+\beta }({\mathbb {R}})^2 \end{aligned}$$

is an isomorphism. We may infer from this property that $(\lambda ^2\partial _p^2+H'^2\partial _q^2,\mathop {\textrm{tr}}\nolimits _0):\mathbb {Y}\rightarrow \mathbb {Z}_1\times \mathbb {Z}_2$ is an isomorphism too. Since

$$\begin{aligned} \Bigg [h\mapsto \Bigg (-3\lambda ^2\gamma H'^2h_p, -(1-\partial _q^2)^{-1}\Bigg [\mathop {\textrm{tr}}\nolimits _0h-\frac{\lambda ^4}{\alpha }\Bigg (S[h]-\int _0^1S[h]\, \textrm{d} q\Bigg )\Bigg ]\Bigg )\Bigg ]:\mathbb {Y}\rightarrow \mathbb {Z}_1\times \mathbb {Z}_2 \end{aligned}$$

is a compact operator, the desired claim for $\partial _h\mathcal {F}(\lambda ,0)$ follows at once. $\square$

The next lemma characterizes the functions that belong to the kernel of $\partial _h\mathcal {F}(\lambda ,0).$

#### Lemma 4.3

Assume that (1.3) is satisfied and set

4.9 $$\begin{aligned} a:=1/H', \end{aligned}$$

where *H* is the unique solution to (4.1). Then, given $\lambda >0$, $h\in \mathbb {Y}$ satisfies $\partial _h\mathcal {F}(\lambda ,0)[h] = 0$ iff $h_0=0$ and for all $1\le k\in {\mathbb {N}}$, we have

$$\begin{aligned} \left. \begin{array}{rllllll} &{}&{} \lambda ^2 (a^3h_k')' -(2k\pi )^2 a h_k = 0 \quad \text {in } \textrm{C}^\beta ([p_0,0]),\\[1ex] &{}&{} \big ( g\lambda ^4 +\alpha (2k\pi )^4 \big )h_k(0) = \lambda ^4a^3(0)h_k'(0),\\[1ex] &{}&{} h_k(p_0)=0, \end{array} \right\} \end{aligned}$$

where the function $h_k\in {\hbox {C}}^{2+\beta }([p_0,0])$, $k\in {\mathbb {N}}$, is defined by

4.10 $$\begin{aligned} h_k(p) := \int _0^1 h(q,p) \cos (2k\pi q) \,\textrm{d}q,\qquad p\in [p_0,0]. \end{aligned}$$

#### Proof

Let $h\in \mathbb {Y}$ satisfy $\partial _h\mathcal {F}(\lambda ,0)[h] = 0$. Then, the relation $L [h]=0$ is equivalent to

$$\begin{aligned} \lambda ^2 h_k''-3\lambda ^2\gamma H'^2h_k' -(2k\pi )^2H'^2h_k = 0 \quad \text {in } {\hbox {C}}^\beta ([p_0,0]) \text { for all } k\in {\mathbb {N}}. \end{aligned}$$

The latter identity is obtained by multiplying the equation $L[h]=0$ by $\cos (2k\pi q)$, followed by integration on ${[p_0,0]}.$ Since *a* is positive and $\gamma =-aa',$ cf. (4.1)_1_, we may reformulate the latter equation as

$$\begin{aligned} \lambda ^2 (a^3h_k')' -(2k\pi )^2 a h_k = 0 \quad \text {in } \textrm{C}^\beta ([p_0,0]) \text { for all } k\in {\mathbb {N}}. \end{aligned}$$

Furthermore, since $h\in \mathbb {Y}$ we have

$$\begin{aligned} h_0(0) = 0, \end{aligned}$$

while, arguing similarly as above, the relation $T[h]=0$ is equivalent to

$$\begin{aligned} \big ( g\lambda ^4 + \alpha (2k\pi )^4 \big )h_k(0) = \lambda ^4 a^3(0) h_k'(0)\qquad \text {for all } k\ge 1. \end{aligned}$$

Finally, since each $h\in \mathbb {Y}$ vanishes on the boundary $p=p_0,$ it holds that

$$\begin{aligned} h_k(p_0)=0\qquad \text {for all } k\in {\mathbb {N}}. \end{aligned}$$

Noticing that the function $h_0\in {\hbox {C}}^{2+\beta }([p_0,0])$ solves the boundary value problem

$$\begin{aligned} (a^3h_0')' = 0 \quad \text {in } [p_0,0],\qquad h_0(0)=h_0(p_0)=0, \end{aligned}$$

it is straightforward to conclude that actually $h_0=0$. This proves the claim. $\square$

In Lemma 4.3, we have shown that a function $h\in \mathbb {Y}$ with $h_0=0$ solves ${\partial _h\mathcal {F}(\lambda ,0)[h] = 0}$ iff for all ${k\ge 1}$ the function $h_k$ defined in (4.10) is a solution to the Sturm–Liouville problem

4.11 $$\begin{aligned} \left. \begin{array}{rllllll} &{}&{} \lambda ^2 (a^3 f')' -\mu a f = 0\quad \text {in } [p_0,0],\\[1ex] &{}&{} ( g\lambda ^4 + \alpha \mu ^2 )f(0) = \lambda ^4 a^3(0) f'(0), \\[1ex] &{}&{} f(p_0) = 0 \end{array} \right\} \end{aligned}$$

with $\mu :=(2k\pi )^2$. We next determine $\lambda _*>0$ such that (4.11) has a nontrivial solution for ${\mu =(2\pi )^2}$ and only the trivial solution $f=0$ when $\mu >(2\pi )^2$. As a first step we show that the solutions to (4.11) build a vector space of dimension less or equal to 1 for each choice of the parameters $\lambda >0$ and ${\mu \in {\mathbb {R}}}$. To this end, we define the Sturm–Liouville-type operator $R_{\lambda ,\mu }:\textrm{C}^{2+\beta }_0([p_0,0])\rightarrow {\hbox {C}}^{\beta }([p_0,0])\times {\mathbb {R}}$ by

4.12 $$\begin{aligned} R_{\lambda ,\mu }[f]:=\begin{pmatrix} \lambda ^2 (a^3 f')' -\mu a f \\[1ex] \lambda ^4 a^3(0) f'(0)-( g\lambda ^4 + \alpha \mu ^2 )f(0) \end{pmatrix}, \end{aligned}$$

where ${\hbox {C}}^{2+\beta }_0([p_0,0]):=\{f\in {\hbox {C}}^{2+\beta } ([p_0,0])\,:\, f(p_0)=0\}$. Let further $f_1,\, f_2\in \textrm{C}^{2+\beta }_0([p_0,0])$ denote the solutions to the initial value problems

4.13 $$\begin{aligned} \left. \begin{array}{lll} \lambda ^2 (a^3 f_1')' -\mu a f_1 = 0\qquad \text {in } [p_0,0],\\[1ex] f_1(p_0)=0,\quad f_1'(p_0)=1, \end{array} \right\} \end{aligned}$$

and

4.14 $$\begin{aligned} \left. \begin{array}{lll} \lambda ^2 (a^3 f_2')' -\mu a f_2 = 0\qquad \text {in } [p_0,0],\\[1ex] f_2(0)= \lambda ^4 a^3(0),\quad f_2'(0)= g\lambda ^4 +\alpha \mu ^2. \end{array} \right\} \end{aligned}$$

#### Lemma 4.4

Given $\lambda >0$ and $\mu \in {\mathbb {R}}$, the operator $R_{\lambda ,\mu }$ is a Fredholm operator of index zero with $\textrm{dim} \ker R_{\lambda ,\mu }\le 1$. Additionally, $\textrm{dim} \ker R_{\lambda ,\mu }= 1$ iff the functions $f_1$ and $f_2$ are linearly dependent. In this case, we have $\ker R_{\lambda ,\mu }=\textrm{span}\{f_1\}.$

#### Proof

Since $[f\mapsto (\lambda ^2 (a^3 f')', \lambda ^4 a^3(0) f'(0))]:{\hbox {C}}^{2+\beta }_0([p_0,0])\rightarrow \textrm{C}^{\beta }([p_0,0])\times {\mathbb {R}}$ is obviously an isomorphism and $R_{\lambda ,\mu }$ is a compact perturbation of this operator, it follows that $R_{\lambda ,\mu }$ is indeed a Fredholm operator of index zero. Moreover, if $f,\,\widetilde{f}\in \ker R_{\lambda ,\mu }$, then $f'\widetilde{f}=f\widetilde{f}'$, which shows that $\textrm{dim} \ker R_{\lambda ,\mu }\le 1$.

Let $\textrm{dim} \ker R_{\lambda ,\mu }= 1$ and let $0\ne f\in \ker R_{\lambda ,\mu }$. Then, since $a^3(ff_i'-f'f_i)$, $i=1,\, 2,$ is a constant function, the initial conditions in (4.13)–(4.14) ensure that this function is in fact identically zero, hence $f_1$ and $f_2$ are linearly dependent. Vice versa, if $f_1$ and $f_2$ are linearly dependent, then they both belong to $\ker R_{\lambda ,\mu },$ and this completes the proof. $\square$

In view of Lemma 4.4, it remains to look for a value $\lambda _*>0$ with the property that the Wronskian ${f_1f_2'-f_1'f_2}$ vanishes in $[p_0,0]$ only for $\mu =(2\pi )^2$. Since $a^3(f_1f_2'-f_1'f_2)$ is a constant function in $[p_0,0]$ and $a\ne 0,$ the Wronskian vanishes in $[p_0,0]$ iff it vanishes at the point $p=0$. Therefore, we consider the function $W:(0,\infty )\times {\mathbb {R}}\rightarrow {\mathbb {R}}$ given by

$$\begin{aligned} W(\lambda ,\mu )=f_1(0)f_2'(0)-f_1'(0)f_2(0)=(g\lambda ^4 + \alpha \mu ^2)f_1(0)-\lambda ^4 a^3(0)f_1'(0). \end{aligned}$$

Observing that (4.13)_1_ depends smoothly on $\mu$ and $\lambda$, this property is inherited also by the solution to (4.13), cf., e.g., [5], and therefore we have $W\in {\hbox {C}}^\infty ((0,\infty )\times {\mathbb {R}}).$

For the special value $\mu =0$, we have

$$\begin{aligned} W(\lambda ,0)=\lambda ^4(g f_1(0)- a^3(0)f_1'(0)). \end{aligned}$$

In view of (4.13), we compute, in the particular case $\mu =0$, that

$$\begin{aligned} f_1'(0)=\frac{a^3(p_0)}{a^3(0)}\qquad \text {and}\qquad f_1(0)=\int _{p_0}^0\frac{a^3(p_0)}{a^3(p)}\textrm{d}p, \end{aligned}$$

which leads to

4.15 $$\begin{aligned} W(\lambda ,0)=\lambda ^4a^3(p_0)\Big (g \int _{p_0}^0 \frac{1}{a^3(p)}\textrm{d}p- 1\Big ). \end{aligned}$$

Hence, if

4.16 $$\begin{aligned} g \int _{p_0}^0\frac{1}{a^3(p)}\textrm{d}p< 1, \end{aligned}$$

then

4.17 $$\begin{aligned} W(\lambda ,0)<0\qquad \text {for all } \lambda >0. \end{aligned}$$

We next investigate the behavior of $W(\lambda ,\mu )$ when $\mu \rightarrow \infty$ .

#### Lemma 4.5

Given $\lambda >0$, it holds that $\lim _{\mu \rightarrow \infty } W(\lambda ,\mu )=\infty .$

#### Proof

Let $m,\, M\in (0,\infty )$ be defined as $m:=\min _{[p_0,0]} a$ and $M:=\max _{[p_0,0]}a$. We note that, since ${f_1'(p_0)=1}$ and ${f_1(p_0)=0}$, there exists $\overline{p}\in (0,p_0]$ such that $f_1(p)>0$ in ${(p_0,\overline{p}).}$ This property together with (4.13)_1_ implies that $a^3f_1'$ is a non-decreasing function in $(p_0,\overline{p})$ and that ${f_1'(p)\ge (m/M)^3}$ for all $p\in [p_0,\overline{p}]$. Consequently, by the fundamental theorem of calculus, $f_1(\overline{p})\ge (\overline{p}-p_0) (m/M)^3>0$ and, therefore, we may actually chose ${\overline{p}=0}$. Thus, $f_1$ is an increasing function in $[p_0,0]$ and $f_1(0)\ge |p_0|(m/M)^3.$

Integrating the first equation of (4.13) over $[p_0,0]$, we have

$$\begin{aligned} a^3(0)f_1'(0)=a^3(p_0)+\frac{\mu }{\lambda ^2}\int _{p_0}^0a(p)f_1(p)\,\textrm{d}p\le M^3+\frac{M|p_0|\mu }{\lambda ^2} f_1(0)\qquad \text {for all } \lambda ,\, \mu \in (0,\infty ). \end{aligned}$$

This estimate together with the definition of $W(\lambda ,\mu )$ and the relation $f_1(0)\ge |p_0|(m/M)^3$ implies that

$$\begin{aligned} W(\lambda ,\mu )\ge (\alpha \mu ^2- \lambda ^2 M|p_0|\mu ) f_1(0)-\lambda ^4 M^3\underset{\mu \rightarrow \infty }{\longrightarrow }\infty , \end{aligned}$$

and the desired claim follows. $\square$

From now on, we assume that (4.16) is satisfied. In view of Lemma 4.5, for each given ${\lambda >0}$, the function $W(\lambda ,\cdot )$ has at least a positive zero. We next investigate the partial derivatives $W_\lambda (\lambda ,\mu )$ and $W_\mu (\lambda ,\mu )$ in all points $(\lambda ,\mu )$ having the property that ${W(\lambda ,\mu )=0.}$

#### Lemma 4.6

Let $\lambda ,\, \mu \in (0,\infty )$ be given such that $W(\lambda ,\mu )=0$. We then have:

(i) $$W_\lambda (\lambda ,\mu )<0$$;
(ii) $$W_\mu (\lambda ,\mu )>0$$ and
  4.18 $$\begin{aligned} \frac{W_\lambda (\lambda ,\mu )}{W_\mu (\lambda ,\mu )}= -\frac{2\mu }{\lambda }. \end{aligned}$$

#### Proof

If $$W(\lambda ,\mu )=0$$, the solutions $$f_1$$ and $$f_2$$ to (4.13) and (4.14) are linearly dependent, hence $$f_1=\Theta f_2$$ with $$ \Theta \in {\mathbb {R}}.$$ By Lemma 4.5, $$f_1 $$ is positive in $$(p_0,0]$$. Recalling that also $${f_2(0)>0}$$, it follows that actually $$\Theta >0$$.

Given $(\lambda ,\mu )\times {\mathbb {R}}\in (0,\infty )$, an application of the chain rule yields that

$$\begin{aligned} W_\lambda (\lambda ,\mu )=(g\lambda ^4 + \alpha \mu ^2)f_{1,\lambda }(0)-\lambda ^4 a^3(0)f_{1,\lambda }'(0) +4\lambda ^3g f_1(0)-4\lambda ^3a^3(0)f_1'(0), \end{aligned}$$

where $f_{1,\lambda }\in {\hbox {C}}^{2+\beta }([p_0,0])$ is the solution to

4.19 $$\begin{aligned} \left. \begin{array}{lll} \lambda ^2 (a^3 f_{1,\lambda }')' -\mu a f_{1,\lambda } = -2\lambda (a^3 f_1')'\qquad \text {in } [p_0,0],\\[1ex] f_{1,\lambda }(p_0)=0,\quad f_{1,\lambda }'(p_0)=0. \end{array} \right\} \end{aligned}$$

We next multiply (4.19)_1_ by $f_1$ and subtract from this relation the identity (4.13) multiplied with $f_{1,\lambda },$ to obtain, after integration on $[p_0,0],$ that

$$\begin{aligned} \lambda a^3(0)f_1(0)f_{1,\lambda }'(0)=\lambda a^3(0)f_1'(0)f_{1,\lambda }(0)-2 a^3(0)f_1(0)f_{1}'(0)+2\int _{p_0}^0a^3(p)f_1'^2(p)\,\textrm{d}p. \end{aligned}$$

If $W(\lambda ,\mu )=0$, the latter relation together with the identity $f_1=\Theta f_2$, $\Theta >0$, leads us to

$$\begin{aligned} \begin{aligned} f_1(0)W_\lambda (\lambda ,\mu )&=2\lambda ^3\Big (2gf_1^2(0) -a^3(0)f_1(0)f_1'(0)-\int _{p_0}^0a^3(p)f_1'^2 (p)\,\textrm{d}p\Big )\\&=2\lambda ^3\Big ( a^3(0)f_1(0)f_1'(0)- \frac{2\alpha \mu ^2}{\lambda ^4}f_1(0)^2- \int _{p_0}^0a^3(p)f_1'^2(p)\,\textrm{d}p\Big ). \end{aligned} \end{aligned}$$

We may now multiply (4.13)_1_ by $$f_1$$ and integrate over $$[p_0,0] $$ to obtain that

4.20 $$\begin{aligned} a^3(0)f_1(0) f_1'(0)=\frac{\mu }{\lambda ^2}\int _{p_0}^0 a(p)f_1^2(p)\,\textrm{d}p+\int _{p_0}^0 a^3(p)f_1'^2(p) \,\textrm{d}p, \end{aligned}$$

hence, on the one hand

4.21 $$\begin{aligned} f_1(0)W_\lambda (\lambda ,\mu )=2\lambda ^3\Big ( \frac{\mu }{\lambda ^2}\int _{p_0}^0 a(p)f_1^2(p)\,\textrm{d}p-\frac{2\alpha \mu ^2}{\lambda ^4}f_1(0)^2\Big ), \end{aligned}$$

and, on the other hand

4.22 $$\begin{aligned} f_1(0)W_\lambda (\lambda ,\mu )= & {} 2\lambda ^3\Bigg (2gf_1^2(0)- 2\int _{p_0}^0 a^3(p)f_1'^2(p) \,\textrm{d}p \nonumber \\{} & {} -\frac{\mu }{\lambda ^2}\int _{p_0}^0 a(p)f_1^2(p)\,\textrm{d}p\Bigg ). \end{aligned}$$

Hölder’s inequality now yields

4.23 $$\begin{aligned} gf_1^2(0)=g\Bigg (\int _{p_0}^0f_1'(p)\,\textrm{d}p\Bigg )^2\le g\Bigg (\int _{p_0}^0\frac{1}{a^3(p)}\,\textrm{d}p\Bigg )\Bigg (\int _{p_0}^0a^3(p)f_1'^2(p)\,\textrm{d}p\Bigg ) \nonumber \\ \end{aligned}$$

and together with (4.22) and (4.16), we have

$$\begin{aligned} \frac{f_1(0)W_\lambda (\lambda ,\mu )}{ 4\lambda ^3}< \Bigg (g\int _{p_0}^0\frac{1}{a^3(p)}\,\textrm{d}p-1\Bigg ) \Bigg (\int _{p_0}^0a^3(p)f_1'^2(p)\,\textrm{d}p\Bigg )<0, \end{aligned}$$

which proves (i).

To prove (ii), we proceed similarly as above and compute, for given ${\lambda ,\,\mu \in (0,\infty )}$, that

$$\begin{aligned} W_\mu (\lambda ,\mu )=(2g\lambda ^4 - \alpha \mu ^2)f_{1,\mu }(0)-\lambda ^4 a^3(0)f_{1,\mu }'(0) -2\alpha \mu f_1(0), \end{aligned}$$

where $f_{1,\mu }\in {\hbox {C}}^{2+\beta }([p_0,0])$ is the solution to

4.24 $$\begin{aligned} \left. \begin{array}{lll} \lambda ^2 (a^3 f_{1,\mu }')' -\mu a f_{1,\mu } = af_1\qquad \text {in } [p_0,0],\\[1ex] f_{1,\mu }(p_0)=0,\quad f_{1,\mu }'(p_0)=0. \end{array} \right\} \end{aligned}$$

We next multiply (4.24)_1_ by $f_1$ and subtract from this relation the identity (4.14) multiplied with $f_{1,\mu },$ to obtain, after integration on $[p_0,0],$ that

$$\begin{aligned} a^3(0)f_1(0)f_{1,\mu }'(0)= a^3(0)f_1'(0)f_{1,\mu }(0)+\frac{1}{\lambda ^2}\int _{p_0}^0a(p)f_1^2(p)\,\textrm{d}p. \end{aligned}$$

Hence, if $W(\lambda ,\mu )=0$, the latter identity combined with the relation $f_1=\Theta f_2$, $\Theta >0$, leads us to

$$\begin{aligned} \frac{\mu f_1(0)W_\mu (\lambda ,\mu )}{\lambda ^4}=-\Bigg ( \frac{\mu }{\lambda ^2}\int _{p_0}^0 a(p)f_1^2(p)\,\textrm{d}p-\frac{2\alpha \mu ^2}{\lambda ^4}f_1(0)^2\Bigg ), \end{aligned}$$

and (ii) follows in view of (4.21) and (i). $\square$

Given $$\lambda >0$$, let

4.25 $$\begin{aligned} \mu (\lambda ):=\inf \{\mu>0:\, W(\lambda ,\mu )>0\}. \end{aligned}$$

Since *W* is smooth, (4.17) and Lemma 4.5 imply that $\mu (\lambda )$ is well defined for all $\lambda >0$ and moreover $\mu (\lambda )>0$. In fact, Lemma 4.6 implies that, for each $\lambda >0,$ $\mu (\lambda )$ is the unique positive zero of the mapping $W(\lambda ,\cdot )$. Moreover, the implicit function theorem together with Lemma 4.6 ensures that

$$\begin{aligned} {[}\mu \mapsto \lambda (\mu )]:(0,\infty )\rightarrow (0,\infty ) \end{aligned}$$

is smooth. We now use the chain rule together with (4.18) to compute that

$$\begin{aligned} \mu '(\lambda )=-\frac{W_\lambda (\lambda ,\mu )}{W_\mu (\lambda ,\mu )}= \frac{2\mu (\lambda )}{\lambda },\qquad \lambda >0, \end{aligned}$$

from where we infer that there exists a positive constant $C_0$ such that

4.26 $$\begin{aligned} \mu (\lambda )=C_0\lambda ^2,\qquad \lambda >0. \end{aligned}$$

It is remarkable that exactly the same expression for $\mu$ (with a possibly different constant $C_0$) has been obtained in [16] in the analysis of the bifurcation problem for stratified capillary-gravity waves. We arrive at the following result.

#### Lemma 4.7

Let

4.27 $$\begin{aligned} \lambda _*:=\frac{2\pi }{\sqrt{C_0}}, \end{aligned}$$

where $C_0$ is the constant identified in (4.26). Then, $\partial _h\mathcal {F}(\lambda _*,0)$ is a Fredholm operator of index zero and with a one-dimensional kernel spanned by

4.28 $$\begin{aligned} h_*(q,p):=f_{1,*}(p)\cos (2\pi q), \quad (q,p)\in \overline{\Omega }, \end{aligned}$$

where $$f_{1,*} $$ denotes the solution to (4.13) corresponding to the parameters $$(\lambda ,\mu )=(\lambda _*,(2\pi )^2).$$

#### Proof

Since $\mu (\lambda _*)=(2\pi )^2$ is the unique positive zero of $W(\lambda _*,\cdot ),$ see (4.25)–(4.27), the claim follows from Lemma 4.2, Lemma 4.3, and Lemma 4.4. $\square$

To apply [14, Theorem 1.7] in the context of the bifurcation problem (4.5), it remains to prove that the transversality condition

4.29 $$\begin{aligned} \partial _{\lambda h}\mathcal {F}(\lambda _*,0)[h_*]\not \in {\text {im}}\partial _{h}\mathcal {F}(\lambda _*,0), \end{aligned}$$

with $\lambda _*$ and $h_*$ introduced in (4.27) and (4.28), is satisfied. Therefore, we first characterize in Lemma 4.8 below the range of $\partial _{h}\mathcal {F}(\lambda _*,0).$

#### Lemma 4.8

A pair $(F,\varphi )\in \mathbb {Z}_1\times \mathbb {Z}_2$ belongs to ${\text {im}}\partial _{h}\mathcal {F}(\lambda _*,0)$ iff

4.30 $$\begin{aligned} \int _\Omega a^3h_*F\,\textrm{d}(q,p)+\alpha C_0(1+C_0\lambda _*^2)\int _0^1 \varphi \mathop {\textrm{tr}}\nolimits _0h_*\,\textrm{d}q=0, \end{aligned}$$

where $h_*\in \ker \partial _{h}\mathcal {F}(\lambda _*,0)$ is defined in (4.28).

#### Proof

As a starting point we observe that for $\partial _{h}\mathcal {F}(\lambda _*,0)=(L,T)$, we have

$$\begin{aligned} L[h]=\lambda _* ^2 a^{-3}(a^3h_p)_p+a^{-2}h_{qq},\qquad h\in \mathbb {Y}. \end{aligned}$$

We now assume that $(F,\varphi )\in {\text {im}}\partial _{h}\mathcal {F}(\lambda _*,0)$ is the image of a function $h\in \mathbb {Y}$. Multiplying the identity $L[h]=F$ by $a^3h_*$ and integrating the resulting relation by parts, we find that

4.31 $$\begin{aligned} \int _\Omega a^3h_*F\,\textrm{d}(q,p)=\int _0^1\lambda _*^2\mathop {\textrm{tr}}\nolimits _0(a^3h_ph_*)\,\textrm{d}q-\int _\Omega \big [\lambda _*^2 a^3 h_p h_{*,p}+ah_qh_{*,q}\big ]\,\textrm{d}(q,p).\nonumber \\ \end{aligned}$$

Moreover, after multiplying the relation $T[h]=\varphi$ by $\mathop {\textrm{tr}}\nolimits _0h_*$ and integrating the resulting relation by parts, we obtain, in view of the symmetry of the operator $(1-\partial _q^2)^{-1},$ that

$$\begin{aligned} \int _0^1\lambda _*^4\mathop {\textrm{tr}}\nolimits _0(a^3h_ph_*)\,\textrm{d}q= & {} \int _0^1(\alpha (2\pi )^4+g\lambda _*^4)\mathop {\textrm{tr}}\nolimits _0( hh_*)\,\textrm{d}q\\{} & {} -\alpha (2\pi )^2(1+(2\pi )^2)\int _0^1 \varphi \mathop {\textrm{tr}}\nolimits _0h_*\,\textrm{d}q. \end{aligned}$$

In virtue of (4.26), (4.27), and (4.13), we have

4.32 $$\begin{aligned} (\alpha (2\pi )^4+g\lambda _*^4)\mathop {\textrm{tr}}\nolimits _0 h_*=\lambda ^4_*\mathop {\textrm{tr}}\nolimits _0(a^3h_{*,p}) \end{aligned}$$

and the latter identity can, thus, be recast as

4.33 $$\begin{aligned} \int _0^1\lambda _*^2\mathop {\textrm{tr}}\nolimits _0(a^3h_ph_*)\,\textrm{d}q=\int _0^1\lambda ^2_*\mathop {\textrm{tr}}\nolimits _0(a^3h_{*,p}h)\,\textrm{d}q-\alpha C_0(1+(2\pi )^2)\int _0^1 \varphi \mathop {\textrm{tr}}\nolimits _0h_*\,\textrm{d}q, \nonumber \\ \end{aligned}$$

where $C_0$ is defined in (4.26). We now sum up (4.31) and (4.33) to conclude that

4.34 $$\begin{aligned}{} & {} \int _\Omega a^3h_*F\,\textrm{d}(q,p)+\alpha C_0(1+(2\pi )^2)\int _0^1 \varphi \mathop {\textrm{tr}}\nolimits _0h_*\,\textrm{d}q \nonumber \\{} & {} \qquad =\int _0^1\lambda ^2_*\mathop {\textrm{tr}}\nolimits _0(a^3h_{*,p}h)\,\textrm{d}q-\int _\Omega \big [\lambda _*^2 a^3 h_p h_{*,p}+ah_qh_{*,q}\big ]\,\textrm{d}(q,p). \qquad \end{aligned}$$

Moreover, arguing as above, but interchanging the roles of *h* and $h_*$, we arrive, in view of ${\partial _{h}\mathcal {F}(\lambda _*,0)[h_*]=0,}$ at

$$\begin{aligned} \begin{aligned}&\int _0^1\lambda ^2_*\mathop {\textrm{tr}}\nolimits _0(a^3h_{*,p}h)\,\textrm{d}q-\int _\Omega \big [\lambda _*^2 a^3 h_p h_{*,p}+ah_qh_{*,q}\big ]\,\textrm{d}(q,p)=0. \end{aligned} \end{aligned}$$

This relation together with (4.34) immediately implies (4.30).

Noticing that (4.30) defines a closed subspace of $\mathbb {Z}_1\times \mathbb {Z}_2$ of codimension 1 which contains the range of ${\partial _{h}\mathcal {F}(\lambda _*,0),}$ the desired claim follows now from Lemma 4.2. $\square$

We are now in a position to verify the transversality condition (4.29).

#### Lemma 4.9

We have $$\partial _{\lambda h}\mathcal {F}(\lambda _*,0)[h_*]\not \in {\text {im}}\partial _{h}\mathcal {F}(\lambda _*,0)$$.

#### Proof

Recalling (4.8), we have

$$\begin{aligned} \begin{aligned} \partial _{\lambda h}\mathcal {F}_1(\lambda _*,0)[h_*]&:=2\lambda _* a^{-3}(a^3h_{*,p})_p,\\ \partial _{\lambda h}\mathcal {F}_2(\lambda _*,0)[h_*]&:= \frac{4\lambda _*^3}{\alpha } (1-\partial _q^2)^{-1}\Big [ S [h_*]-\int _0^1S[h_*]\, \textrm{d} q\Big ], \end{aligned} \end{aligned}$$

where, using the definition of the operator *S* and that of $h_*$ together with (4.32), we have

$$\begin{aligned} S [h_*]-\int _0^1S[h_*]\, \textrm{d} q=-\frac{1}{(2\pi )^2}\mathop {\textrm{tr}}\nolimits _0(a^3h_{*,p}-gh_*)=-\frac{\alpha C_0^2}{(2\pi )^2}\mathop {\textrm{tr}}\nolimits _0 h_*. \end{aligned}$$

Appealing to (4.20), we compute

$$\begin{aligned} \int _\Omega a^3h_* \partial _{\lambda h}\mathcal {F}_1(\lambda _*,0)[h_*]\,\textrm{d}(q,p)&=\lambda _*\Big (a^3(0)f_{1,*}(0)f_{1,*}'(0)-\int _{p_0}^0 a^3(p)f_{1,*}'^2(p)\,\textrm{d}p\Big )\\&=\lambda _*\Big ((g+\alpha C_0^2)f_{1,*}^2(0) -\int _{p_0}^0 a^3(p)f_{1,*}'^2(p)\,\textrm{d}p\Big ) \end{aligned}$$

and

$$\begin{aligned} \alpha C_0(1+C_0\lambda _*^2)\int _0^1 \partial _{\lambda h}\mathcal {F}_2(\lambda _*,0)[h_*]\mathop {\textrm{tr}}\nolimits _0h_*\,\textrm{d}q&=2\lambda _*f_{1,*}(0)(a^3(0)f_{1,*}'(0)-2gf_{1,*}(0))\\&=-2\alpha \lambda _* C_0^2f_{1,*}^2(0), \end{aligned}$$

hence, using also (4.23) and (4.16), we get

$$\begin{aligned}&\int _\Omega a^3h_* \partial _{\lambda h}\mathcal {F}_1(\lambda _*,0)[h_*]\,\textrm{d}(q,p)+\alpha C_0(1+C_0\lambda _*^2)\int _0^1 \partial _{\lambda h}\mathcal {F}_2(\lambda _*,0)[h_*]\mathop {\textrm{tr}}\nolimits _0h_*\,\textrm{d}q\\&\qquad =\lambda _*\Big ((g-\alpha C_0^2)f_{1,*}^2(0) -\int _{p_0}^0 a^3(p)f_{1,*}'^2(p)\,\textrm{d}p\Big )\\&\qquad<\lambda _*\Big (gf_{1,*}^2(0) -\int _{p_0}^0 a^3(p)f_{1,*}'^2(p)\,\textrm{d}p\Big )\\&\qquad \le \lambda _*\Big ( g \int _{p_0}^0\frac{1}{a^3(p)}\,\textrm{d}p-1\Big )\int _{p_0}^0 a^3(p)f_{1,*}'^2(p)\,\textrm{d}p<0. \end{aligned}$$

This proves the claim. $\square$

We are now in a position to establish Theorem 1.1.

#### Proof of Theorem 1.1

In view of Lemma 3.1 and of Proposition 2.1, in the framework of waves which are symmetric with respect to the vertical line $x=0$, the Euler formulation (1.2) of the steady hydroelastic waves problem is equivalent to the height function formulation (3.8), hence also to the bifurcation problem (4.5). Therefore, the assertion (i) is a straightforward consequence of Lemma 4.1.

Concerning (iia), if (1.4) is not satisfied, then $W(\lambda ,0)\ge 0$ for all $\lambda >0,$ see (4.15), and Lemmas 4.5 and 4.6 (ii) then ensure that $W(\lambda ,\mu )>0$ for all $\mu >0$. Lemmas 4.2, 4.3, and 4.4 then imply that $\partial _h\mathcal {F}(\lambda ,0)$ is an isomorphism, hence $(\lambda ,0)$ is not a bifurcation point for (4.5), regardless of the value of $\lambda >0$.

It remains to establish (iib). Therefore, we note that (4.15), (4.16), Lemmas 4.5, and 4.6 (ii) imply that for each $\lambda >0$, the nonlinear equation $W(\lambda ,\cdot )=0$ has a unique solution $\mu =\mu (\lambda )>0$, which is given by (4.26). Our previous requirements that the boundary value problem (4.11) has a nontrivial solution for $\mu =(2\pi )^2$ and only the zero solution for $\mu >(2 \pi )^2$ identifies a unique value $\lambda _*$, see (4.27), with this property. The smoothness property (4.6) together with Lemmas 4.7 and 4.9 enable us now to use the local bifurcation theorem due to Crandall and Rabinowitz, cf. [14, Theorem 1.7], in the context of (4.5), to conclude, in view of the equivalence of the formulations (1.2) and (4.5), the existence of the smooth local bifurcation curve

4.35 $$\begin{aligned} {[}s\mapsto (\lambda (s),h(s))]:(-\varepsilon ,\varepsilon )\rightarrow (0,\infty )\times \mathcal {O}, \end{aligned}$$

where $\varepsilon >0$ is small, such that $\mathcal {F}(\lambda (s),h(s))=0$ for all $|s|<\varepsilon$. Moreover, $\lambda (0)=\lambda _*$ and

4.36 $$\begin{aligned} h(s)=s(h_*+\chi (s)), \end{aligned}$$

where $\chi \in {\hbox {C}}^\infty ((-\varepsilon ,\varepsilon ),\mathbb {Y})$ satisfies $\chi (0)=0$. Arguing similarly as in [13, Section 5], it is not difficult to prove that, since the function $h_*$ satisfies ${h_*(q,0)=f_{1,*}(0)\cos (2\pi q),\, q\in {\mathbb {R}}}$, see (4.28), with $f_{1,*}(0)>0$, also the waves profile $\eta (s)$ has for ${s\ne 0}$ exactly one maximum (at $x=0$) and minimum (at $x=\lambda (s)/2$) per period. This completes the proof. $\square$

## Data Availability

Not applicable.

## Footnotes

1 Note that if $(u,v,P,\eta )$ is a solution to (1.2), then for each $c>0$ the tupel $(\widetilde{u},\widetilde{v},\widetilde{P},\widetilde{\eta }):=(u-c,v,P,\eta )$ is a hydroelastic wave solution traveling with wave speed constant *c*.
